# Do photographs, older adults’ narratives and collaborative dialogue foster anticipatory reflection (“preflection”) in medical students?

**DOI:** 10.1186/s12909-016-0802-2

**Published:** 2016-11-11

**Authors:** Gabrielle Brand, Ashlee Osborne, Mark Carroll, Sandra E. Carr, Christopher Etherton-Beer

**Affiliations:** 1 Education Centre, the University of Western Australia, Faculty of Medicine, Dentistry & Health Sciences, M515, 35 Stirling Hwy, Crawley, WA 6009 USA; 2 Western Australian Centre for Health & Ageing, Centre for Medical Research and School of Medicine and Pharmacology, University of Western Australia; Geriatrician and Clinical Pharmacologist, Royal Perth Hospital, Perth, Australia

## Abstract

**Background:**

In changing higher education environments, medical educators are increasingly challenged to prepare new doctors to care for ageing populations. The Depth of Field: Exploring Ageing resource (DOF) uses photographs, reflective questioning prompts, older adults’ narratives and collaborative dialogue to foster anticipatory reflection or ‘preflection’ in medical students prior to their first geriatric medicine clinical placement. The aim of this research is to explore whether photographs, narratives and small group collaborative dialogue fosters reflective learning, enhances reflective capacity and has the potential to shift medical students’ attitudes towards caring for older adults.

**Methods:**

This study used a mixed method evaluation design, measuring attitudes using pre and post questionnaire responses and individual written reflections drawn from 128 second year medical students, exploring their perceptions toward older adults.

**Results:**

Quantitative and qualitative data indicated that the DOF session generated reflective learning that resulted in positive shifts in medical students’ perceptions towards older adults. The qualitative reflections were captured in four main themes: the opportunity provided to *Envision* working with older adults; the *Tension* created to challenge learners’ misinformed assumptions, and the work of *Dismantling* those assumptions, leading to *Seeing* older people as individuals.

**Conclusions:**

These findings highlight how visual and narrative methodologies can be used as an effective reflective learning tool to challenge medical students’ assumptions around ageing and how these may influence their care of older adults.

## Background

How can we better prepare future doctors to care for ageing populations? Medical educators must be responsive to the changing demographics of population ageing, as older adults are one of the main health care consumer groups [1]. The need for medical students to become reflective learners and practitioners is of paramount importance [2–4], with an increasing focus on *learning processes* (as opposed to just *gaining knowledge* [5]). Reflective learning is integral in enhancing critical analysis of both experience and knowledge to ensure future health professionals are self-aware and engage in self-monitoring of their ongoing professional practice [6].

A recent study assessing the attitudes of medical students has found students to have moderately negative attitudes towards older adults, as well as little expressed desire to work with them [7]. Teal et al. [8] notes that reflective activities, such as writing, small group discussion, imagery exercises and perspective taking exercises, are used within medical education to surface stereotypical views and unconscious biases. In addition, socializing medical students with older adults in an intergenerational arts program [9] and narratives using doctor and patient stories, literature and film have also been successfully used to promote more patient or relationship focused care [10]. Photographs of older adults have also been used as an effective way to generate reflection amongst nursing students, encouraging them to anticipate their clinical placement in an aged care setting in more meaningful ways [11].

Reflective practitioners, [12] actively reflect during (reflection-in-action) and after (reflection-on-action) an event. Reflection-before-action, described as thinking through intentions before one proceeds [13] or anticipatory reflection [14], is also increasingly recognised as being important. This ‘preflection’ allows one to consider possible outcomes and anticipate experience before an event. Despite the educational importance of *preflection*, there is limited published literature on educational innovations and/or teaching tools and strategies that use *preflection* to address attitudes toward certain patient groups [8]. In particular, we found no research that explicitly evaluates *preflection* aiming to raise learners’ awareness and expose hidden stereotypes in an attempt to counteract disinterest in caring for older adults prior to geriatric medicine clinical practice; which will be the focus of this study.

## Methods

A mixed method evaluation design was applied to explore three research questions:

1. What are the perceptions of a group of 2^nd^ year medical students towards caring for older adults prior to their first geriatric clinical placement?
2. How do photographs, narratives and small group collaborative dialogue (Depth of Field: Exploring Ageing ©) influence reflective learning in medical students?
3. What effect does this reflective learning technique have on medical students’ attitudes towards caring for older adults?

### Description of Depth of Field: Exploring Ageing © (DOF)

The DOF reflective learning resource was developed in response to previous pilot qualitative research that explored the use of photo-elicitation techniques, older adults’ narratives and collaborative dialogue in the classroom to enhance reflective learning experiences by surfacing unconscious bias in health professions students’ perceptions towards older adults [15]. The main aim of the resource is to stimulate in-depth discussion and challenge health professions students through reflection to consider new perspectives around ageing that move ‘beyond diagnosis’ to a more humanistic model of care for older people [16]. The learning outcomes for DOF include students’ being able to:

- Identify and examine their personal perceptions of older persons.
- Express their views to their peers in a shared reflective learning journey.
- Explore and reflect on their role as a health profession student working with older people.

A short background on reflection and instructions on constructing a shared narrative through collaborative dialogue precede students (in groups of 3 or 4) being shown a series of five photographs accompanied by reflective questioning prompts. No prior context is given to the students. At the end of the session, students are shown a 3 min, audio-narrated film of the older adult featured in the photographs to challenge any unexplored assumption or (mis-) conceptions around ageing. This aims to surface any cognitive discrepancy between what the students thought they knew, and the older person’s *actual* narrative, creating the tension required for transformational learning [17] including shifts in perspectives, to occur. The session is concluded with a full-group facilitated guided discussion.

### Structure of reflective learning sessions

The University runs a graduate entry 4 year medical program. In their 2nd year, medical students enrol in a semester-long, Integrated Medical Practice unit which involves several clinical attachments including a four week Geriatric Medicine rotation. A key learning outcome of this rotation is for medical students to demonstrate a positive approach to older age and illness, including an awareness of ageism and negative stereotypes of older persons that persists in today’s society.

### Recruitment

Prior to their geriatric medicine rotation, students were invited to participate in a 1 h DOF reflective learning session and to submit a 500 word written reflection within four weeks. Eight, one hour standardised sessions were conducted by the first author over a period of 5 months in semester 2, 2015 with groups ranging from six students to 21 students. Altogether, 128 students (out of a cohort of 240) attended the sessions and 95 students submitted an individual written reflection. The study obtained university ethics approval and all participants completed a written consent prior to commencing the DOF session.

### Data collection

Participants completed a pre-questionnaire (before the DOF reflective learning session) that included age, gender, previous work with or close personal relationships with an older adult and a 13-item validated Geriatric Attitude Scale (adapted with permission by Reuben et al. 1998 [18]. This tool was designed to measure several key constructs including general impressions and perceived value of older people, potential benefits of treating older patients, distributive justice of resource use, and economic concerns about caring for older people. It was chosen as it is a short instrument that measures general attitudes of primary care residents towards older people, including attitudes towards caring for older patients and demonstrates high reliability with a Cronbach’s alpha of 0.76 [18]. Following the DOF session, students completed the same questionnaire as before the session (post-questionnaire) and were instructed to submit an individual 500 word written reflection within 4 weeks.

### Data analysis

All data were de-identified prior to analysis. Descriptive statistics were applied to the demographic data including gender and age. A non-parametric Wilcoxon Signed-Ranks Test was used to compare an item’s response distribution on the post-questionnaire with its response distribution on the pre-questionnaire. To allow for multiple comparisons, probability values less than 0.01 were regarded as significant. Effect size was calculated following Kerby [19] as a matched-pairs rank-biserial correlation. Thresholds for assessing effect size followed Cohen (2002), i.e. *r* = 0.10 for Small Effect, *r* = 0.30 for Medium Effect and *r* = 0.50 for Large Effect.

We acknowledge the conflict of interest that may exist in evaluation of qualitative findings from a resource that was developed by two authors on this paper. Therefore, steps were undertaken to reduce any potential bias [20], and maintain trustworthiness of the study findings. These included qualitative data analysis being conducted independently by the first author, and by two qualitative independent coders who were not involved in the development of the resource. The research team discussed ideas and key concepts that stood out from the data, emerging patterns, threads and themes were assigned colour codes. Manual coding was used as a method of analysing the large amount of qualitative data. This was part of the analytical process because giving codes to data and developing concepts, enabled a rigorous evaluation of what the data was saying. This process continued until all the data was coded as saturation was not reached until the bulk of the written reflections had been analysed and coded. The next stage involved exploring, playing with and linking created codes and concepts [21] until consensus between authors was reached and distilled into four main themes.

## Results

### Characteristics of participants

Of the 128 participants, 55 % were female and 45 % were male and 83 % were aged in the 20–25 or 26–30 age brackets with the remainder being older. Gender and age distributions of the participants were not significantly different to those of the medical student cohort from which they were drawn. The majority of participants (65 %) indicated that they had a close relationship with a person aged 70 or older and in most of these cases (88 %) they rated that relationship as “Fulfilling” or “Very Fulfilling”. The majority (61 %) of participants indicated that they had previously worked with older adults.

### Attitudes towards older adults

Table 1 indicates the comparison between 128 participants’ paired responses to the questionnaire completed before the reflective learning session and to the same questionnaire completed immediately after the session. For each item, the (baseline) percentage of participants who responded positively to the item on the pre-questionnaire is also shown. For several of the items on the pre-questionnaire (i.e. items 1, 3, 9, 10, 11 and 13), responses were mostly positive. However, for other items the number of positive pre-responses was more modest (items 4, 6 and 12) and for a few items on the pre-questionnaire (items 2, 5, 7 and 8), responses were mostly negative or neutral.

**Table 1:** Overall shift in participants’ responses (*n* = 128) from the pre to the post questionnaire geriatric attitude scale (adapted with permission by Reuben, et al., 1998 [18]

Item	Pre-test % positive responses	│Z│^a^	*p*	Shift in responses	Effect size
1. Most older people are pleasant to be with	84	2.819	<0.01	Positive	Medium (*r* = 0.49)
2. If I have the choice, I would rather see younger patients than older ones	27	5.317	<<0.01	Positive	Large (*r* = 0.76)
3. It is society’s responsibility to provide care for older persons	89	0.333	>0.01	Not Significant	
4. Medical care for older people uses up too much human and material resources	63	2.629	<0.01	Positive	Medium (*r* = 0.38)
5. As people grow older, they become less organized and more confused	27	6.017	<<0.01	Positive	Large (*r* = 0.80)
6. Older patients tend to be more appreciative of the social work care I provide than are younger patients	63	1.178	>0.01	Not Significant	
7. Taking a medical history from older patients is frequently an ordeal	27	3.126	<0.01	Positive	Medium (*r* = 0.46)
8. I tend to pay more attention and have more sympathy towards my older patients than my younger patients	26	4.241	<<0.01	Positive	Large (*r* = 0.65)
9. Older people in general do not contribute much to society	85	2.135	>0.01	Not Significant	
10. Treatment of chronically ill older patients is hopeless	95	1.658	>0.01	Not Significant	
11. Older persons don’t contribute their fair share towards paying for their health care	84	2.652	<0.01	Positive	Medium (*r* = 0.43)
12. In general, older persons act too slowly for modern society	77	1.762	>0.01	Not Significant	
13. It is interesting listening to older people’s accounts of their past experiences	85	3.865	<<0.01	Positive	Large (*r* = 0.62)

^a^Wilcoxon Signed Ranks Test

Following the DOF session, there was a significant overall positive shift in responses for eight of the 13 items. That is, the response distribution for each of these items was significantly more positive on the post-questionnaire than on the pre-questionnaire. For the eight significant items, effect sizes were large for half of the shifts and medium for the remainder. Results were non-significant for other items. No items showed a negative shift. Items that showed a large positive effect size were mostly those which displayed a low (baseline) percentage of positive responses on the pre-questionnaire, such as items 2, 5 and 8 around preferences for younger patients and a belief that older people become less organized and more confused. Most of the items that showed no significant shift in response distribution displayed a comparatively high (baseline) percentage of positive responses on the pre-questionnaire, such as items 3, 9 and 10 around the contribution that older people make to society and the responsibility and value of providing care for older persons.

### Qualitative themes

A total of 95 of the 128 participants (74 %) submitted a written reflection, 51,715 words were analysed and generated the following four major themes – Envisioning; Creating Tension; Dismantling Assumptions, and Seeing the Person.

### Envisioning “…I think I will feel…”

For most of the medical students, the opportunity to *preflect* provided a safe space, a starting point to begin to anticipate and envision how they might feel in their future geriatric medicine clinical placement:

*Examining my thoughts approaching the commencement of my geriatrics placement I realized I was mentally dragging my feet, and not looking forward to the coming experiences….*

This experience brought up a wide range of emotive responses including feeling: ‘apprehensive’, ‘daunted’, ‘uncomfortable’, ‘sad’, ‘confronting’, ‘challenging’, ‘incompetent’, ‘ambivalent’, ‘excited’, ‘frustrated’, and ‘overwhelmed’. Interestingly, many of the students experienced a mix of emotions as they anticipated their geriatric placements:

*Sometimes I imagine I would feel sad and helpless upon viewing someone who is decrepit and terminal, and other times I might feel happy and surprised when an older adult has a strong spirit and a bright sense of humour.*

Although the pre versus post-questionnaire data indicated that the medical students experienced some positive shifts in their perceptions towards caring for older adults prior to their clinical placement, the discourse used in many of the individual written reflections suggests that they perceived older adults as experiencing a significant decrease in quality of life and high levels of disease and disability – mainly due to physical and cognitive impairment and chronic diseases.

For many of the students a perceived inability to treat or cure their older patients (therapeutic nihilism) was a concern as they wrote of the suffering and poor prognosis for this group and an expressed sadness at the inevitability of death:

*I expected to feel saddened by the situations older patients were presented with, namely chronic illnesses, multiple co-morbidities, cognitive impairment, isolation, lack of independence and loneliness. I also expected to feel a sense of hopelessness when faced with these issues and the inevitability of death and dying.*

This was extended by another group of students who in their written text included passive, paternal, and patronizing and/or pejorative terms such as “so cute”, “jovial”, “I like old people”, and “old people are frail but they are very lovely to work with”.

Many of the medical students also expressed concern around aspects of communication – with many citing ‘history taking’ within limiting ‘time constraints’ as a source of anticipated concern and challenge, particularly with regard to how this might affect their clinical practice:

*I am conscious of the fact that some older adults are not the greatest historians, and I wonder how this will affect my ability to take a medical history and form a differential diagnosis from the information obtained.*

### Creating tension: “I never imagined that…”

A major theme identified in the majority of medical student reflections was that tension occurred following the DOF session which many described as a “surprise” on two different levels. First, they were surprised at what the older person can actually do/what they are capable of (a contradiction to what they had assumed) and second, were surprised at themselves as many students admitted feeling uncomfortable when their personal assumptions of older people were contradicted by the older adult’s actual story (presented at the end of the session). For example:

*There was my assumption that older adults are technologically illiterate but here was this older adult playing a game that I don’t even know how to play!*

A number of students displayed awareness that they were using their assumptions and stereotypes to ‘fill the gaps’ in the imagined story:

*It is easy to make assumptions when you don’t have much information to work with.*

While for others, it felt uncomfortable as one student recounts her experience of creating her narrative:

*The group and myself were somewhat reluctant to give an answer at first, and I think this was because it felt wrong to make generalisations about a person you knew next to nothing about. Eventually, with encouragement, we participated in the session. It was at this time that our latent perceived generalisations came to the fore. Of course, nothing bad was said, but it was interesting to note that something had to be said – and it was our preconceptions that filled these voids.*

Interestingly, many students reported discomfort with this type of teaching methodology with some reporting that having their assumptions surfaced brought up “uncomfortable” and/or “guilty” feelings:

*After the session, I felt slightly guilty and uncomfortable having jumped to conclusions about what the older adult can and cannot do. These feelings, however, have undoubtedly contributed to me learning to shift my perceptions, be more open to possibilities and more importantly to not assume in whatever I do.*

### Dismantling assumptions: “…all the older people seemed happier than they should be”

A shared theme in the majority of the written reflections was that this reflective learning methodology surfaced the students’ assumptions around ageing:

*Many of my assumption about older adults were challenged, and some of these assumptions I wasn’t even aware I was making until today.*

Based on the written reflection, it was from close and fulfilling personal relationships with an older person (usually grandparents) that many had based their preconceptions and stereotypes of older people. For most, this was a positive relationship for others negative, but the reflective learning was powerful as many revealed how this relationship had influenced their perceptions of older adults and disease processes:

*Dementia really disturbs me – I think this is due to my experiences with my grandmother who died from Alzheimer’s disease.*

Previous work with older people was a characteristic shared by 61 % of this cohort which for some was an acknowledged source of preconceived bias towards older adults:

*I have cared for older patients who are at both ends of the spectrum – grumpy, demanding and rude; through to those who feel like a burden and don’t want to bother anyone, thus neglecting their own health. I know that this may cause me to form assumptions based on past experiences, and it forced me to recall my attitude and demeanor towards each of those patients. I can recall times that I have treated patients poorly because of first impressions (e.g. they were filthy dirty and reeked of alcohol) and this may have led to suboptimal care on my part. …….I can adjust my attitude to be more open and receptive.*

Another reflection included recognition of how the media plays an influence on assumptions and belief systems around ageing:

*Our assumptions of older people were challenged.....From a young age we have been exposed to films and newspaper articles which form a preconceived idea about the elderly. Often older adults are portrayed as frail, slow, mentally impaired and in physical decline …*

Recognising, naming and sharing their assumptions with peers provided the space, time and permission to question where their assumptions and sometimes unconscious belief systems came from as one student wrote the act of “expressing it makes it real” and another relays:

*This experience will encourage me to spend more time reflecting on my feelings on clinical placements …and I see the value in sharing my experiences and perspectives with my peers. I was surprised how much I was able to learn through communicating with the group, and getting constructive feedback.*

Most of the medical students reported feeling surprised at hearing their peers’ contrasting assumptions. The students found it interesting that each member of the group interpreted the photographs differently. For example, some reported a look of sadness on the man’s face, whereas others saw a look of contentment. Some felt extreme sympathy at the photo of the man attempting to fit his prosthetic leg, whereas others identified with the man’s stoicism. Interestingly, this ‘emotional’ reading of the same photos ranged from positive (‘content’, ‘satisfied’, ‘joyful’, ‘happy’, ‘passionate’) to negative (‘lonely’, ‘sad’, ‘traumatized’, ‘haunted’, ‘struggling’). The students also found value in learning to dialogue with one another:

*The most pertinent point I learnt from this exercise was the differing interpretation and assumptions amongst our peer group. …….I guess that this shows that perspective can dramatically change the way an older adult is perceived, and that it is not too much of a stretch to realise that this could impact on the way that they are treated by health professionals and the level of health care they are given.*

### Seeing the person: “I learnt to never judge a book by its cover”

For many students a key outcome of the DOF session was recognition that older people ‘are just people too’. This is best summed up by the comment one student made: “older adults were once regular people just like you and me”. This recognition was a surprise for some with a number admitting that their previous perception of older adult patients had been very negative.

*….seeing the pictures of an elderly man playing games on his computer and building a model battleship, and then having to form a narrative around those pictures, evoked a rather intense internal dialogue - I realised that he was just a normal person with hobbies and interests who happens to be old, rather than an ‘old person’.*

For one student this recognition was described simply as “…I often forget that not every older person is having a miserable time”. This humanizing of older people was displayed as many of the students began to recognise older adults as individuals rather than a homogenous group:

*I learned it was not appropriate to consider elderly under a one-size fits all approach.*

*In reality there were two narratives. There was the story of who this man was…and there was his medical history. The two were inherently related but the later did not detract from the former. He is who he is and then, later in life, he happened to have some medical issues. The disease-or disability-does not define the person, or reshape their personal narrative.*

However, for some students, the session challenged their assumptions but did not necessarily change them:

*My assumptions of older adults were challenged, but not necessarily changed. I am still leaning towards thinking he is an exception rather than a rule…..I think it is sometimes necessary to assume until proven otherwise.*

A key aim of the DOF session is for students to have the opportunity to *preflect* and surface any unexplored assumptions around ageing. Being mindful of the potential impact of unconscious biases was an important and potent learning experience as illustrated in the following quotes:

*This narrative gave me a whole new perspective on how to look at older adults. Age is not a barrier to life, our thoughts are. Each individual has their own story, and it is important for us as future doctors to connect to these individuals and understand their story without passing our judgements unnecessarily.*

For a number of students, the reflective tension produced valuable insights as many began to translate this new awareness and knowledge into how previously held assumptions may influence their future care of older adults:

*Overall, I was amazed to realise just how much of myself, my past experiences and my expectations I can project onto others when I am lacking context. This has been a valuable experience for me in terms of learning about myself. I often feel that these reflective learning experiences are not particularly useful, but I feel that the use of photographs, and the fact they are real people involved, helped elicit the emotional aspect of reflective learning that if often lacking in such activities in the somewhat sterile atmosphere of a university course.*

This reflective learning process provided a valuable learning experience that many identified informing their future practice with older adults:

*…had I not gone through this reflective process, I may have neglected to ask certain questions of patients, due to my assumptions. I may have knowingly filled in the gaps of what they have told me, creating what I believe to be their story and not what is truly their story.*

Students also translated this learning into other medical specialties and began to focus on older people’s ability rather than disability:

*…it has become evident that a lot of them are usually a lot more capable than what can be assumed about them. It is these stereotypes that can negative impacts in clinical practice as it can lead to biased opinions and affect the doctor-patient relationship. Therefore I hope to be able to apply this not only to elderly patients, but to all patients that I come across in my career.*

## Discussion

These results suggest that Depth of Field: Exploring Ageing © reflective learning resource successfully uses photographs, narratives and collaborative dialogue to assist students to identify and examine their perceptions towards older adults, express these views to their peers and anticipate their role as a medical student prior to their first clinical experience in geriatric medicine.

In this study, before the reflective learning session, participating medical students reported a variety of attitudes towards older adults. Responses to items on the pre-questionnaire, varied from mostly positive through moderately positive to mostly negative or neutral. Likewise, the individual written reflections demonstrated a diverse range of perceptions and expectations toward working with older people with many students expecting/anticipating low quality of life and high levels of disability and mortality in their older patients. This finding may be a contributing factor to the widespread disinterest in medical students choosing geriatrics as a career [7] which research has shown can often stem from inaccurate perceptions around aspects of health care [22] and negative stereotypes and societal obsession with youth [23]. The findings are consistent with studies that have found residents with dementia self-reported quality of life scores are higher than those reported by nursing staff [24, 25].

In this study, a positive shift in perceptions of medical students towards caring for older adults following the DOF session was evident in both quantitative and qualitative data sets. Post-questionnaire data indicate that these positive shifts were generally strongest in areas where prior attitudes were the least positive, such as preferences for seeing younger rather than older patients, and the belief that people become less organized and more confused as they grow older. Students’ written reflections suggest that the surfacing, sharing and challenging of negative assumptions and stereotypes around older people and ageing through the DOF session provided a valuable reflective learning experience which could positively inform their future practice with older patients. This is supported by Sandars [3] who claims reflection should be used in medical education to develop therapeutic relationships as it illuminates unconscious interactions (influenced by beliefs and values) which can ultimately affect decision making and action that impact patient outcomes.

Collectively, the findings suggest a widening perspective and growing sense of empathy as a result of the DOF session that encouraged recognition and active deconstruction and sharing (through collaborative dialogue and reflective writing) the learners’ perceptions of older adults, particularly in regard to being more open to engaging with older adults (over younger patients) and the value of listening to older adults’ past experiences. Overwhelmingly, the majority of medical students were surprised at how this reflective learning methodology prompted an uncomfortable tension or as Wear and Aultman [26] term “pedagogy of discomfort” that invites learners to actively identify, question and address any unconscious bias or prejudices which we believe is all part of the intellectual and emotional work of deep reflection that precedes what Freire ([27] p. 27) describes as a “re-reading of the world”. The findings demonstrate the importance of facilitating learning opportunities for *preflection* as they create a safe space for students to begin the developmental process of becoming aware of potential bias, to explore and unpack their thoughts around ageing and integrate this growing awareness into their future clinical experiences and practise with older adults in more mindful ways. Seeing the older adult, as an individual and being able to recognize previously held assumptions and work toward mitigating the influence (of these unconscious biases) on their interactions with older adults [8] during their future clinical experiences is essential in cultivating reflective practitioners that maintain a high standard of professional practice [28].

While this study affirms the use of visual and narrative methodologies as a potent reflective learning tool in medical education, it must be acknowledged that it is only a snapshot of the perceptions of one group of medical students towards older adults and those students’ reflective learning experiences. Further research would be necessary with other health profession students to determine if the findings are consistent and transferable to other student populations. However, this research reveals and offers some key insights into how we might begin to integrate reflective learning methodologies into medical curriculum, in particular to explore the transformative potential of fostering *preflection* in medical students and the subsequent impact it may have on their perceptions towards older adults. Educational research that explores the interplay between photographs, narrative pedagogy and collaborative dialogue and how it creates safe learning spaces that foster individual, collective and transformative reflective learning also warrants further exploration.

## Conclusions

In a time of demographic changes and growing older adult populations, it is vital that we respond to workforce changes by producing patient centered medical practitioners that adopt positive perceptions towards caring for older adults. To ensure that this occurs, it is imperative that we create reflective learning spaces in medical education curriculum for students to practise reflection. As found in this research, this can be achieved by implementing simple visual and narrative methodologies that engage students in the process of reflective learning, which includes the important transformative work of analyzing, critical questioning, dismantling and ultimately reframing perceptions towards older adults.

## Abbreviation

DOF: Depth of Field: Exploring Ageing resource
